# Modulatory effects of dietary prickly pear (*Opuntia ficus-indica*) peel on high salinity tolerance, growth rate, immunity and antioxidant capacity of Nile tilapia (*Oreochromis niloticus*)

**DOI:** 10.1007/s10695-023-01289-z

**Published:** 2024-01-05

**Authors:** Mohamed E. Salem, Hebatollah M. Almisherfi, Abdel-Fattah M. El-Sayed, Sarah O. Makled, Heba M. Abdel-Ghany

**Affiliations:** 1 National Institute of Oceanography and Fisheries, NIOF, Cairo, Egypt; 2 Oceanography Department, Faculty of Science, Alexandria University, Alexandria, Egypt

**Keywords:** Prickly pear, Nile tilapia, Salinity stress, Growth performance, Anti-oxidation, Innate immunity

## Abstract

This study evaluated the effects of prickly pear (*Opuntia ficus-indica*) peel (PPP) on salinity tolerance, growth, feed utilization, digestive enzymes, antioxidant capacity, and immunity of Nile tilapia (*Oreochromis niloticus*). PPP was incorporated into four iso-nitrogenous (280 g kg^−1^ protein) and iso-energetic (18.62 MJ kg^−1^) diets at 0 (PPP0), 1 (PPP1), 2 (PPP2), and 4 (PPP4) g kg^−1^. Fish (9.69 ± 0.2 g) (mean ± SD) were fed the diets for 75 days. Following the feeding experiment, fish were exposed to a salinity challenge (25‰) for 24 h. Fish survival was not affected by the dietary PPP inclusion either before or after the salinity challenge. Fish fed the PPP-supplemented diets showed lower aspartate aminotransferase, alanine aminotransferase, cortisol, and glucose levels compared to PPP0, with the lowest values being observed in PPP1. Fish fed dietary PPP had higher growth rates and feed utilization than PPP0. Quadratic regression analysis revealed that the best weight gain was obtained at 2.13 g PPP kg^−1^ diet. The highest activities of protease and lipase enzymes were recorded in PPP1, while the best value of amylase was recorded in PPP2, and all PPP values were higher than PPP0. Similarly, PPP1 showed higher activities of lysozyme, alternative complement, phagocytic cells, respiratory burst, superoxide dismutase, glutathione peroxidase and catalase, and lower activity of malondialdehyde than in PPP0. Further increases in PPP levels above 2 g kg^−1^ diet led to significant retardation in the immune and antioxidant parameters. Thus, the inclusion of PPP at about 1 to or 2 g kg^−1^ diet can improve stress tolerance, immunity, and antioxidant capacity in Nile tilapia.

## Introduction

Seawater intrusion into natural rivers is one of the effects of sea level rise caused by climate change (Agoubi 2021). As a result, freshwater salinization has become a global and growing problem affecting both biodiversity and ecosystems (Canedo-Argüelles et al. 2013). The activity, physiological performance, and composition of fish hormones and enzymes, as well as their survival and behavior are affected by salinity (Wang and Zhu 2002). On the other hand, tilapia is currently the most widely farmed fish group in the world, second only to carp, with a production of 6.1 million mt in 2020, valuing over 12 billion US$ in freshwater and brackishwater environments (FAO 2022). These fish are characterized by their fast growth, reproduction capacity, physiological strength, potent resistance to stress and diseases, adaptability to different environmental conditions, and trophic plasticity (El-Sayed 2020a). Despite the fact that tilapia are euryhaline, which  can tolerate a wide range of water salinity (El-Sayed 2020b), they also show a significant increase in Na^+^ , K^+^ -ATPase activity in the gill, plasma osmolality, cortisol, glucose, growth hormone and prolactin after exposure to salinity stress (Fiess et al. 2007; Breves et al. 2010; Angadi et al. 2021; Sallam et al. 2020). Moreover, the physiological changes associated with salinity acclimation in tilapia are energy-demanding (Morgan et al. 1997; Angadi et al. 2021). This indicates that these fish may encounter biological and physical alterations as a result of salinity stress (Fiess et al. 2007; Kammerer et al. 2010). Therefore, mitigating the impacts of salinity stress is crucial for maintaining optimal growth and physiological functions in farmed tilapia. Formulating immunostimulant- and antioxidant-rich diets may boost tilapia health and production, and strengthen their ability to overcome stress.

There is an increasing public awareness and safety concern regarding the use of synthetic immune stimulants and antioxidants in aquaculture, due to their impacts on human health and environment, in addition to their potential adverse effects on fish physiology and immunity (Shah et al. 2014; Yamashita et al. 2009). Therefore, the search for natural alternatives has become necessary. In this regard, fruit and vegetable wastes and by-products have attracted the attention as cheap potential health stimulants, due to the natural biomolecules they contain (Acar et al. 2015; Baba et al. 2016; Acar et al. 2018; Kesbiç and Yigit 2019; Van Doan et al. 2021a, b, c).

Prickly pear (*Opuntia ficus-indica*) (PP) (locally known as Barbary fig) is a widely distributed fruit in arid and semi-arid regions all over the world and has been used in traditional medicine for decades in the treatment of a number of diseases such as ulcer, diabetes, dyspnea, liver diseases, glaucoma, wounds, and fatigue (Hegwood 1990; Livrea and Tesoriere 2004; Dhaouadi et al. 2013). Prickly pear is an important source of active elements such as total phenols, carotenoids, flavonoids, betalain, linoleic acid, taurine, vitamins (C, group B, E and carotene), minerals (calcium, potassium, phosphorus and selenium) and free amino acids (phenylalanine, proline, alanine, histidine and lysine) (Ghazi et al. 2015). These elements are indispensable for human health (Hfaiedh et al. 2008) because they exhibit antioxidant, anti-inflammatory, anti-allergic, neuro-protective, and anticancer properties (El-Hawary et al. 2020; Benayad et al. 2014). PP cladode extracts may also reduce serum cholesterol levels and blood pressure (Agozzino et al. 2005). Moreover, their generated by-products during fruit processing, including seed, pulp and peel, contain large amounts of high-value bioactive compounds (Barba et al. 2017; Milán-Noris et al. 2016). PP extracts are also able to upregulate heat shock proteins, thereby providing protection against pathogen infection and other environmental stressors in aquatic organisms (Boerrigter et al. 2014; Ravishankar et al. 2023; Sung et al. 2012).

Despite these attributes, limited information is available on the use of PPP as a functional feed additive in aquaculture. As far as the authors know, only a single study evaluated the effects of supplemental PPP, at high concentrations (10–20%), on the performance and health status of Nile tilapia (Ahmed et al. 2020). However, it is not known whether lower levels of PPP would improve fish growth and health parameters. Therefore, this study was carried out to investigate the effects of low concentrations of PPP on salinity stress tolerance, growth performance, feed utilization, antioxidant activity and immune response of Nile tilapia (*O. niloticus*) juveniles.

## Materials and methods

### Fish and experimental conditions

This study was carried out at Al‐Max Research Station, the National Institute of Oceanography and Fisheries, Alexandria, Egypt. Nile tilapia (*O*. *niloticus*) juveniles were obtained from a local commercial tilapia hatchery. The fish were randomly distributed, in triplicates, into 12 glass aquaria (70 L) filled with dechlorinated tap water, at a density of 10 fish aquarium^−1^. Each aquarium was aerated through an air compressor. The fish were acclimated to the culture conditions for 2 weeks; during the first week, they were fed a commercial tilapia diet (28% CP), while during the second week, they were fed the test diets. After the acclimation period, all fish in each aquarium were counted, weighed collectively, and the average initial weight was recorded, which was 9.69 ± 0.20 g fish^−1^ (mean ± SD). The aquaria were cleaned daily, and feces were siphoned before the first feeding. The siphoned water was replaced by aerated de-chlorinated water from a storage tank. The water quality parameters, including temperature, dissolved oxygen (DO), pH and total ammonia (NH_4_), were measured weekly, using Hanna Aquaculture Multi-parameter Photometer, HI83303. The average values of these parameters throughout the experiment were 25 ± 1.9 °C, 6.5 mg L^−1^, 7.7, and 0.06 mg L^−1^, respectively. The fish were subjected to a natural lighting cycle.

### Experimental diets and feeding regime

Four iso-nitrogenous (28% cp), iso-energetic (18.62 MJ kg^−1^) diets were produced, containing four levels of dried prickly pear fruit (*Opuntia ficus-indica*) peel (PPP) (0, 1, 2, and 4 g kg^−1^ diet), designated as PPP0 (control), PPP1, PPP2, and PPP4, respectively (Table 1). The PPP used in this study was obtained from a local market in Alexandria, Egypt. The fresh peels were oven-dried at 60 °C for 48 h and ground into fine particles using a laboratory grinder (Tornado, MG-2000, China). Diet ingredients were weighed and mixed together with warm distilled water until stiff dough was obtained. The diets were then passed through a meat grinder to form spaghetti-like threads, spread on aluminum foil plates, and sun-dried outdoors for 48 h. The dried diets were finally mashed (2.5 mm), labeled and stored in plastic bags at − 20 °C until used.

**Table 1:** Formulation and proximate analysis of the test diets fed to *O. niloticus*

Ingredients (g kg^−1^)	Diet 1 (PPP0)	Diet 2 (PPP1)	Diet 3 (PPP2)	Diet 4 (PPP4)
Fish meal (700 g kg^−1^ CP)	50	50	50	50
Soybean meal (440 g kg^−1^ CP)	490	490	490	490
Corn flour	380	380	380	380
Minerals and Vitamins^1^	20	20	20	20
Prickly pear fruit peel powder	0	1	2	4
Starch	10	9	8	6
Oils (50% fish oil:50% corn oil)	40	40	40	40
Di-calcium phosphate	10	10	10	10
Proximate analysis (%)				
Protein	28.10	28.03	28.1	28.23
Ether extract	6.50	6.74	6.76	6.89
Ash	6.49	6.46	6.48	6.50
Fiber	4.48	4.53	4.57	4.64
Gross energy (MJ kg^−1^ DM) dDm)	18.60	18.60	18.55	18.63

^1^Vitamin and minerals mixture contains IU kg^−1^ or mg kg^−1^ of dry vitamins and minerals powder): Vit. A, 2,200,000 IU; Vit. D3, 1,100,000 IU; Vit. E, 1,500 IU; Vit. K, 800; Vit. B1, 1,100; Vit. B2, 200; Vit. B6, 2,000; Vit. H, 15; Vit. B12, 4; Vit. C 3,000; iron, 160; magnesium, 334; copper, 21.6; zinc, 21.6; selenium, 25; cobalt, 2.38

The diets were fed to the respective aquaria, to satiation, three times a day, 6 days a week for 75 days. At the end of the feeding trial, the fish in each aquarium were netted, counted, weighed collectively, and their average weights were recorded. Three fish from each aquarium were randomly selected and frozen at − 20 °C for final body composition analysis.

### Blood sampling

Clove oil (0.1 ml L^−1^) was used as an anesthetic agent during the blood collection (Fernandes et al. 2017). Blood samples (1 mL) were collected from the caudal veins in plastic microtubes, containing dipotassium ethylenediaminetetraacetic acid (EDTA) as an anticoagulant, for hematological tests, using heparinized syringes (Cal heparin 5000 IU, Amoun Co, Egypt). One milliliter of blood, without anticoagulant, was also collected and centrifuged at 2500 × g for 10 min. for serum separation, to be used in the biochemistry assay.

### Acute salinity challenge

After the feeding trial, the remaining seven fish in each aquarium were subjected to a salinity challenge. The salinity of the fish aquaria were raised from 0 to 25‰ over 10 h (about 2.5 degrees every hour) by phasing out fresh water and replacing it with well water (40‰) obtained from a nearby saltwater well. Then, fish were left in this salinity for 24 h and the cumulative mortality was recorded at the end of the salinity challenge. After the salinity challenge, fish were anesthetized with clove oil (0.1 mL L^−1^) (Fernandes et al. 2017) and blood samples  were collected.

### Growth performance and feed utilization

Growth performance and feed utilization were calculated as follows (De Silva and Anderson 1995):

- weight gain (WG) (g) = final weight (g) − initial weight (g),
- average daily gain (ADG) (g) = final weight (g) -initial weight (g) /trial duration (days),
- specific growth rate (SGR) (% day^−1^) = 100 (ln final weight (g) —ln initial weight (g))/trial duration (days),
- feed conversion ratio (FCR) = dry feed intake (g)/live fish WG (g), and
- protein efficiency ratio (PER) = fish live WG (g)/ dry protein intake (g).

### Analyses

#### Body composition and feed analysis

The body proximate composition (moisture, crude protein, ether extract and ash) analyses were determined using the standard methods of AOAC (2009). The moisture content was measured by drying in an oven (105 °C for 24 h). The crude protein content was determined by the Kjeldahl method (Kjeldahl System, K358/355, BUCHI, Flawil, Switzerland). Ether extract (EE) was measured by the solvent extraction method (Soxhlet System- VELP Scientific a, SER 248, Italy). Ash content was determined by incinerating fish samples in a muffle furnace at 600 °C for 6 h.

#### Digestive enzyme analysis

In order to evaluate the effect of PPP on the digestive enzymes activity in Nile tilapia, the fish were anesthetized using clove oil (about 0.5 ml L^−1^), euthanized, dissected under aseptic conditions, and their intestines were carefully removed. The total intestinal contents were collected, rinsed, homogenized and centrifuged at 5000 g for 30 min at 4 °C. The supernatant was recovered and kept at 4 °C for enzymatic assays that were conducted within 24 h after extraction. Protease, amylase and lipase were analyzed according to the standard methods as described by Sun et al. (2011), and their activities were expressed as specific activities (U mg^−1^ protein). Protease activity was determined using the bovine serum albumin standard. Amylase activity was conducted using a starch substrate, whereas lipase activity was measured using β-naphthyl caprylate substrate.

#### Analysis of immunological and antioxidant parameters

Lysozyme (Lyz) activity was analyzed by adding 50 μL of blood serum to 950 μL *Micrococcus lysodeikticus* (Jiancheng, Nanjing, Jiangsu, China) suspension (200 mg mL^−1^), then mixed with 0.05 M sodium phosphate buffer (pH 6.2) at 25 °C (Sun et al. 2010). Optical density (OD) was measured at 530 nm using a spectrophotometer (UV2802S, Shimadzu, Kyoto, Japan). One unit of Lyz is equal to the amount of enzyme producing a decrease in absorbance of 0.001 min^−1^ mL^−1^. Alternative complement activity (ACH50) was analyzed by mixing 0.5 mL of a serially diluted serum with 0.2 mL of erythrocyte suspension (2 × 10^7^ cells mL^−1^), then incubated at 20 °C and pH 7.0 for 2 h in 10 mM EGTA and 10 mM MgCl_2_. Afterward, 1.4 mL of gelatin veronal buffer (GVB) and 10 mM EDTA were added to stop the hemolytic reaction. OD was measured at 414 nm. One unit of ACH50 is equal to the volume of serum complement producing 50% hemolysis (Cheng et al. 2007). Phagocytic activity (PA) was detected by inoculating 200 mL of leucocyte suspensions with 100 μL of formalin-killed *Staphylococcus aureus* ATCC 25923 (the Naval Medical Research Unit 3, Cairo, Egypt) in phosphate-buffered saline (PBS) at room temperature for 30 min. Thereafter, the mixture was centrifuged at 3,000 g for 5 min at 4 °C. Pellets were collected, smeared and stained with Wright Giemsa solution (Sigma-Aldrich, St. Louis, MO, USA). Phagocytic cells were counted microscopically (Sun et al. 2010). Respiratory burst (RB) was detected spectrophotometrically for the formazan produced from the nitroblue tetrazolium (NBT)-O_2_ redox reaction (Franco et al. 2017). One-unit of RB is equal to 0.001 increase in absorbance at 630 nM min^−1^.

Catalase activity (CAT) was analyzed using 3,5-dichloro-2-hydroxybenzene sulfonic acid to rapidly terminate the degradation reaction of hydrogen peroxide (Hamed et al. 2020). Serum superoxide dismutase (SOD) activity was analyzed by a reagent kit (Jiancheng, Nanjing, Jiangsu, China) based on the superoxide reduction of nitroblue tetrazolium (NBT). One unit of SOD activity is equal to the amount of enzyme necessary to produce 50% inhibition of the NBT reduction rate. Optical density was measured with a spectrophotometer (UV-2802S, Shimadzu, Kyoto, Japan) at 550 nm (Sun et al. 2010). Glutathione peroxidase (GPx) was determined by the standard method as described by Khan et al. (2016). The optical density of NADPH was measured at 340 nm and 37 °C and expressed as GPx units (min^−1^ mg^−1^ protein). Malondialdehyde (MDA) was detected calorimetrically, based on thiobarbituric acid reactive substances (TBARS), as described by Ashouri et al. (2015).

#### Blood biochemistry

Liver function enzymes, namely aspartate aminotransferase (AST) and alanine aminotransferase (ALT), were determined colorimetrically by atomic absorption spectrophotometry using the Crest Biosystems® kits according to the manufacturer’s instructions and measured at 505 nm (Akter et al. 2018). Cortisol level was measured by enzyme-linked immunosorbent assay (EIA), while glucose was detected using commercial kits (Spinreact SA, Sant Esteve d'en Bas, Girona, Spain) adapted for 96-well microplates, as detailed by Varela et al. (2010).

#### Statistical analysis

The obtained results were subjected to one-way analysis of variance (ANOVA) at a 95% confidence limit, using the SPSS software, version 23. In addition, two-way ANOVA was used to assess the effects of dietary treatments and salinity challenge on fish physiological functions. Duncan’s multiple range test was used to compare means when *F*-values from the ANOVA test were significant (*P* < 0.05). Simple linear and nonlinear regressions were also carried out to correlate the relationships between measured parameters and supplemental PPP levels.

##### Declaration

All methods are reported in accordance with the ARRIVE guidelines 2.0.

## Results

### Acute salinity challenge

The effects of dietary PPP on the response of Nile tilapia challenged with a salinity stress are summarized in Table 2. Fish transferred from fresh water to 25‰ water salinity over a 10-h period suffered from high mortality, but fish survival was not affected by dietary PPP supplementation *(P* > 0.05). ALT, AST and glucose have been affected by both the experimental diets and salinity. At both salinities, fish fed the PPP-supplemented diets showed significantly lower AST, ALT, cortisol and glucose levels (*P* < 0.05) than the control group, with lowest values being observed at PPP1. However, Nile tilapia fed PPP above 1 g kg^−1^ showed a gradual increase in the concentrations of ALT, AST, cortisol and glucose (*P* < 0.05), but their values were still significantly lower than the control groups (*P* < 0.05).

**Table 2:** The effects of diet on the response of Nile tilapia challenged with high salinity stress

Parameter	FW treatment						Post salinity challenge (25‰)							
	PPP0	PPP1	PPP2	PPP4		PPP0			PPP1		PPP2			PPP4
Survival (%)	90.00 ± 0.00	90.00 ± 0.00	96.67 ± 5.77	93.33 ± 5.77		33.38 ± 4.9			40.00 ± 10.00		40.66 ± 6.81			42.66 ± 2.51
ALT (U mg^−1^ protein)	54.80 ± 0.31	39.07 ± 0.20	40.53 ± 0.49	46.56 ± 0.20		89.81 ± 0.28			42.98 ± 0.37		46.94 ± 1.47			52.23 ± 1.96
AST (U mg^−1^ protein)	90.68 ± 0.14	62.69 ± 0.31	68.16 ± 0.17	88.39 ± 0.59		99.27 ± 1.28			65.24 ± 0.60		70.96			97.52 ± 0.66
Cortisol (µg dL^−1^)	38.54 ± 0.21	31.44 ± 0.29	33.32 ± 0.38	36.75 ± 0.26		87.17 ± 1.84			53.88 ± 1.37		51.82 ± 0.60			56.92 ± 1.68
Glucose (mg dL^−1^)	100.68 ± 0.82	86.98 ± 0.79	88.20 ± 1.35	95.86 ± 0.67		226.27 ± 2.38			95.97 ± 1.42		135.07 ± 0.96			175.60 ± 3.25
Two-way ANOVA														
Variation source	Salinity	Diet	Interaction		Duncan HSD for dietary PPP									
					PPP0			PPP1		PPP2		PPP4		
Survival (%)	***	ns	ns		a			a		a			a	
ALT (U mg^−1^ protein)	***	***	***		a			d		c			b	
AST (U mg^−1^ protein)	***	***	***		a			d		c			b	
Cortisol (µg dL^−1^)	***	***	***		a			c		c			b	
Glucose (mg dL^−1^)	***	***	***		a			d		c			b	

SDM pooled standard deviation of the mean. All values are mean of three independent biological replicates (n = 3). *P < 0.05, **P < 0.01, ***P < 0.001, ns non-significant Significant differences are indicated by different letters (*P* < 0.05)

### Growth performance and feed utilization efficiency

**Fig. 1:**
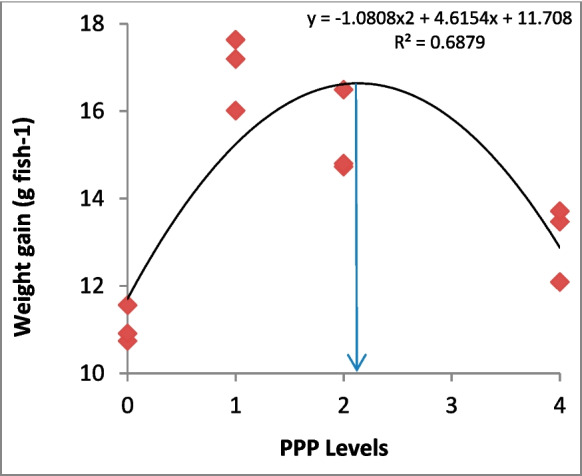
Second-degree polynomial regression of the weight gain of Nile tilapia juveniles fed on the test diets

The present results showed that Nile tilapia fed on dietary PPP had higher growth rates (*P* < 0.05) than the control group (PFP0) (Table 3). PPP1 and PPP2 groups showed the greatest final weight, followed by PFP4, while the lowest weight gain (*P* < 0.05) was found in the PFP0 group. Similar trends were also recorded with regard to feed utilization efficiency (*P* < 0.05). However, quadratic regression analyses indicated that the best growth rates and feed efficiency were obtained at about 2.13 g PPP kg^−1^ diet (Fig. 1). There was no significant difference in survival rates (*P* ˃ 0.05) among the experimental treatments (Table 2).

**Table 3:** Growth performance and feed utilization of Nile tilapia (*O. niloticus*) juveniles fed on the test diets

Parameter	Control (PPP 0)	PPP1	PPP2	PPP4
Initial weight (g fish^−1^)	9.70 ± 0.04	9.70 ± 0.11	9.71 ± 0.27	9.63 ± 0.28
Final weight (g fish^−1^)	20.77 ± 0.46^c^	26.64 ± 0.90^a^	25.05 ± 1.08^a^	22.72 ± 1.05^b^
Gain (g fish^−1^)	11.07 ± 0.43^d^	16.94 ± 0.84^a^	15.34 ± 1.00^b^	13.09 ± 0.87^c^
ADG (g fish^−1^)	0.15 ± 0.01^d^	0.23 ± 0.11^a^	0.20 ± 0.01^b^	0.17 ± 0.01^c^
SGR (% day^−1^)	1.01 ± 0.02^d^	1.35 ± 0.04^a^	1.26 ± 0.05^b^	1.14 ± 0.04^c^
Feed intake (g fish^−1^)	20.38 ± 0.91	20.03 ± 0.62	20.34 ± 1.54	20.46 ± 1.11
FCR	1.84 ± 0.10^a^	1.18 ± 0.02^c^	1.33 ± 0.03^c^	1.57 ± 0.14^b^
PER	1.94 ± 0.11^d^	3.02 ± 0.06^a^	2.69 ± 0.11^b^	2.27 ± 0.21^c^

Data are presented as mean ± SD. Data in the same row with different superscripts are significantly different (*P* < *0.05*). All values are means of three replicates (*n* = 3)

*WG,* weight gain; *ADG,* average daily gain; *SGR,* specific growth rate; *FCR,* feed conversion ratio; *PER,* protein efficiency ratio

### Body composition

The body composition of Nile tilapia fed the test diets is summarized in Table 4. The results demonstrated that PPP-supplemented diets resulted in significantly higher body protein (*P* < 0.05) than the PPP-free (PPP0) diet. However, increasing dietary PPP from 1 to 4 g kg^−1^ did not alter body protein (*P* > 0.05). On the other hand, body lipid, ash and dry matter were not significantly affected by dietary PPP concentrations (*P* > 0.05).

**Table 4:** Chemical body composition (%) of Nile tilapia (*O. niloticus*) juveniles fed on the experimental diets

Parameter	PPP0	PPP1	PPP2	PPP4
Dry matter	32.33 ± 0.83	33.62 ± 1.03	33.71 ± 2.64	32.83 ± 2.17
Crude protein	57.24 ± 1.43^b^	59.03 ± 0.58^a^	59.43 ± 0.59^a^	59.77 ± 0.84^a^
Crude lipid	25.06 ± 0.73	24.80 ± 1.00	23.91 ± 1.47	23.25 ± 0.65
Ash	15.43 ± 0.66	15.90 ± 0.94	15.56 ± 0.70	15.43 ± 0.49

Data are presented as mean ± SD. Data in the same row with different superscripts significantly differ (*P* < 0.05). All values are means of three replicates (*n* = 3)

### Digestive enzymes

The highest activities of digestive protease and lipase were recorded in PPP1 followed by a significant decrease (*P* < 0.05) at 2 g kg^−1^ and 4 g kg^−1^. The best value of amylase activity was recorded in PPP2, followed by PPP1 and PPP3. However, digestive enzyme activities were still higher at all PPP levels than in the control group (Table 5).

**Table 5:** Digestive enzymes activity (U mg^−1^ protein) of Nile tilapia (*O. niloticus*) juveniles fed on the experimental diets

Parameter	PPP0	PPP1	PPP2	PPP4
Protease	8.05 ± 0.10^d^	12.43 ± 0.21^a^	12.00 ± 0.30^b^	9.71 ± 0.35^c^
Lipase	6.40 ± 0.14^c^	11.00 ± 0.10^a^	8.54 ± 0.11^b^	6.70 ± 0.21^c^
Amylase	3.80 ± 0.22^c^	7.00 ± 0.14^a^	6.50 ± 0.35^a^	5.43 ± 0.15^b^

Data are presented as mean ± SD. Data in the same row with different letters significantly differ (*P* < 0.05). All values are mean of three replicates (*n* = 3)

### Immunological and oxidative stress responses

The immunological responses in Nile tilapia were significantly affected by dietary PPP (*P* < 0.05) (Table 6). Lysozyme activity, ACH50 and RB were significantly increased (*P* < 0.05) in fish fed PPP1, but significantly decreased (*P* < 0.05) with further increase in dietary PPP to 2 and 4 g kg^−1^. PA also increased (*P* < 0.05) with increasing dietary PPP to 1 and 2 g kg,^−1^ and significantly decreased at 4 g kg^−1^.

**Table 6:** Serum immunity parameters of Nile tilapia (*O. niloticus*) juveniles fed the test diets

Parameter	PPP0	PPP1	PPP2	PPP4
Lysozyme (U mg^−1^ protein)	4.04 ± 0.15^c^	7.60 ± 0.25^a^	6.70 ± 0.21^b^	4.40 ± 0.20^c^
ACH50 (units ml^−1^)	19.71 ± 0.24^c^	26.45 ± 0.13^a^	23.60 ± 0.23^b^	23.20 ± 0.30^b^
PA (U mg^−1^ protein)	35.64 ± 0.10^d^	42.05 ± 0.30^a^	42.70 ± 0.36^a^	38.50 ± 0.24^c^
RB (%)	1.30 ± 0.20^c^	2.83 ± 0.32^a^	2.40 ± 0.20^b^	2.10 ± 0.10^b^

Data are presented as mean values ± standard deviation of the mean. Data in the same row with different letters significantly differ (*P* < 0.05). All values are means of three replicates (*n* = 3)

Similar responses were observed in the antioxidant capacity in fish fed the test diets (Table 7). The activities of SOD and GPx increased with supplemental PPP to 1 g kg^−1^, but significantly decreased (*P* < 0.05) with further increase in dietary PPP to 2 and 4 g kg^−1^. The values of CAT significantly increased with increasing PPP levels up to 2 g kg^−1^ (*P* < 0.05), then decreased in the PPP4 group. On the other hand, the MDA activity showed an opposite trend, where the values significantly decreased with increasing PPP to 1 g kg^−1^, then increased (*P* < 0.05) with further PPP supplementation.

**Table 7:** Antioxidant parameters of Nile tilapia (*O. niloticus*) juveniles fed on the test diets

Parameter	PFP0	PFP1	PFP2	PFP4
CAT (U mg^−1^)	0.12 ± 0.04^c^	1.74 ± 0.10^a^	2.00 ± 0.10^a^	1.14 ± 0.10^b^
SOD (U mg^−1^)	14.60 ± 0.30^d^	23.35 ± 0.40^a^	21.32 ± 0.20^b^	18.00 ± 0.30^c^
GPx (μmol mg^−1^)	1.00 ± 0.10^c^	3.66 ± 0.33^a^	3.00 ± 0.10^b^	2.54 ± 0.30^b^
MDA (U mg^−1^)	2.50 ± 0.15^a^	0.35 ± 0.03^d^	0.55 ± 0.03^c^	1.04 ± 0.12^b^

Data are presented as mean ± standard deviation of the mean. Data in the same row with different letters significantly differ (*P* < 0.05). All values are means of three replicates (*n* = 3)

## Discussion

Salinity affects physiological performance, composition of the hormones and enzymes, survival, and behavior of fish (Wang and Zhu 2002). In the present study, salinity challenge has elevated the liver enzymes (AST and ALT) and stress indicators (cortisol and glucose) of Nile tilapia (*Oreochromis niloticus*). In support, transferring *O. niloticus* from fresh water to sea water for 2 weeks led to a significant increase in liver enzyme activity (Vijayan et al. 1996). Elevating these enzymes indicates injury of the fish tissues and liver (Asztalos and Nemcsok 1985). Following the same pattern, cortisol and glucose increased in Nile tilapia (*O. niloticus*) with increasing salinity, and survival was affected by higher salinity levels. (Zidan 2022). In a similar way, when the salinity increased to 15 g L^−1^, AST and ALT of common carp (*Cyprinus carpio*) were higher compared to the control group (Al-Khshali and Al Hilali 2019). The concentration of plasma cortisol, which is a key factor in osmoregulation in tilapia (Morgan et al. 1997; Kammerer et al. 2010) sharply increased following the salinity challenge.

In turn, the inclusion of PPP in fish diets in the current study downregulated negative effects of salinity stress in terms of AST, ALT, and cortisol. The level of serum glucose, which is an energy source used in minimizing the adverse effects of salinity stress (Angadi et al. 2021), was also reduced in fish fed PPP. These findings suggest that the inclusion of PP in the tilapia diet can mitigate elevated stress, presumably due to the presence of polyphenols in PP. The total phenolic content for PPP was estimated as 520 ± 2.64 mg gallic acid g^−1^, whereas flavonoids were estimated as 65.7 ± 3.41 mg catechin equivalents g^−1^ (Abdel-Razek et al. 2019). It was reported that inclusion of the polyphenol-rich algae *Laurencia obtusa* in the red tilapia diet relieved hypoxia stress effects by reducing the levels of serum cortisol and glucose (Salem et al. 2021a, b). Moreover, this improvement in the resistance of Nile tilapia to salinity stress by dietary supplementation of PPP may be due to the presence of pinellic acid and quercetin in PPP, which act as anti-inflammatory agents (Aruwa et al. 2019; Durazzo et al. 2021). In support, Nile tilapia fed *Aspergillus oryzae*-supplemented diets and exposed to salinity levels showed a significant decrease in glucose, cortisol, ALT, and AST compared to fish fed on the control diet and challenged with the same salinity levels (Shukry et al. 2021).

In the current study, Nile tilapia fed PPP above 1 mg kg^−1^ showed a gradual increase in the concentrations of ALT, AST, cortisol and glucose, but their values were still significantly lower than the control groups. This may be attributed to the accumulation of anti-nutritional factors present in PP polyphenols such as tannins. The present results are in agreement with Zhong et al. (2020) who documented that high dietary tea polyphenol concentration led to a significant increase of ALT, AST, serum cortisol and glucose levels in juvenile black carp *Mylopharyngodon piceus*. The authors attributed that to the anti-nutritional factors of tea polyphenols which might alter the stress regulation system of the fish and hinder their hepatic metabolism.

The present study demonstrated that dietary PPP, even at low levels, significantly stimulated the growth performance, feed utilization efficiency and crude protein content of Nile tilapia. These results may be due to the phenolic compounds present in PPP, namely gallic and ferulic acids (Dhaoudia et al. 2013; Benayad et al. 2014), which are renowned as growth promoters (Cai et al. 2020; Dawood et al. 2020; Van Doan et al. 2021c; Xu et al. 2021). The improvement of the feed utilization efficiency and body protein content in the present study may be attributed to the ability of dietary PPP to stimulate the digestive enzymes secretion and promote the intestinal microbiota function to digest the nutrients and improve their absorption (Salem et al. 2019; Van Doan et al. (2021b). The increase in digestive enzyme activity in fish fed PPP-supplemented diets in the current study may support this assumption. Dietary PPP in the present study improved the digestive enzymes secretion in Nile tilapia, presumably due to the presence of Cu, Fe, and Zn in *O. ficus-indica* (Bakar et al. 2020), which can promote the activity of protease, lipase and amylase (Li et al. 2007; 2009). Moreover, endophytic fungi associated with *O. ficus indica*, such as isolate PF103, *Acremonium terricola*, *Phoma tropica*, and *Tetraploa aristate*, are good protease stimulator (Bezerra et al. 2012; Wangkahart et al. 2022) which may stimulate the secretion of proteases. The presence of α-amylase enzyme in *O. ficus indica* may have also increased the amylase activity in the present study, as suggested by Ishurd et al. (2010), thereby leading to improved dietary carbohydrate utilization which may have spared dietary protein for somatic growth (Kumar et al. 2006a,b).

In the present study, growth performance and feed utilization decreased with increasing supplemental PPP above 2 g kg^−1^. This could be due to the inability of fish to digest the non-starch polysaccharides present in prickly pear peel (Salem and Abdel-Ghany 2018; Tawfik et al. 2022). The anti-nutritional factors (e.g., phytate, tannin and oxalate), which are considerably high in *O. ficus indica*, may have also adversely affected the growth performance and feed utilization when PPP was supplemented above 2 g kg^−1^ diet (Reda and Atsbha 2019).

The fish innate immune system is the first line of defense against infections. Innate immune parameters, such as lysozyme, alternative complement activity (ACH50), phagocytosis (PA) and respiratory burst (RB), have been used as indicators for the response of fish against stress and disease (Saurabh and Sahoo 2008). In the current study, dietary PPP improved lysozyme activity, ACH50, PA and RB in Nile tilapia. This may be attributed to the presence of bioactive compounds such as flavonoids, polyphenols, vitamin C, and fatty acids, particularly linoleic acid in PPP, which can promote the activity of the above-mentioned immune response parameters (Ahmed et al. 2020; Daniloski et al. 2022; Van Doan et al. 2021b; Kumari and Sahoo 2005).

In the current study, the activity of immune parameters decreased at dietary PPP levels exceeding 1 g kg^−1^. This may be attributed to the increase of polyphenols levels with increasing PP in the diet which might cause the decrease in immune parameters activity and weaken the immune system (Banavreh et al. 2019). It should be mentioned, however, that even at high PPP concentrations, the immune response was still better than the control treatment. Similar results were reported by other authors, where high levels of fruit peels caused adverse effects on farmed fish. For example, Zhuo et al. (2021) found that low levels of lemon peel (1–3%) enhanced the activity of lysozyme in Asian sea bass (*Lates calcarifer*), while higher levels (5%) resulted in poor feed utilization and decreased lysozyme activity. Also, when pomegranate peel was supplemented in common carp (*Cyprinus carpio*) diets at 5 g kg^−1^, it improved the immune parameters and hepatic antioxidant enzymes (Yousefi et al. 2023). At higher concentrations (15 and 20 g kg^−1^), the immune and antioxidant capacities were suppressed.

Antioxidant parameters, such as CAT, SOD, MDA and GPx are the first defense line against oxidative stress (Kasote et al. 2015; Ighodaro and Akinloye 2018). A considerable antioxidant capacity of *O. ficus indica* (43–95% of inhibition) has been documented (Reda and Atsbha 2019). The current results revealed that Nile tilapia fed PPP-supplemented diets exhibited higher antioxidant capacity than fish fed the control diet. This may be because PPP contains significant amounts of natural antioxidants such as vitamins E and C, tannins, carotenoids, polyphenols and betalain (Daniloski et al. 2022; Melgar et al. 2017) which have the ability to stimulate the production of antioxidant enzymes in fish (Ahmed et al. 2020; Salem et al. 2019). The antioxidant activity of Nile tilapia tended to decrease with increasing PPP level in the diet. This may be attributed to the increase of vitamins C and E concentrations with increasing supplemental PPP level. It was reported that increasing dietary levels of vitamins C and E induced oxidative stress in juvenile Japanese flounder (*Paralichthys olivaceus*) (Gao et al. 2014). High concentrations of these vitamins render erythrocyte membranes susceptible to peroxidation and lead to the accumulation of hydroperoxides as a result of lipid peroxidation (Welker and Congleton 2009; Celada et al. 2013). The presence of high amount of phytate in *O. ficus indica* (Reda and Atsbha 2019), which increased with the increase of dietary PP level, may have also reduced the antioxidant capacity (da Costa et al. 2021).

In conclusion, dietary PPP improved tolerance to salinity stress. growth performance, feed utilization, body crude protein, digestive enzymes activity, non-specific immunity and antioxidant activity of juvenile Nile tilapia. However, survival rate was not significantly affected by the PPP inclusion. Therefore, supplementation of PPP, at about 1 or 2 g kg^−1^ diet, can be considered a growth promoter, immunostimulant and anti-stress agent for farmed Nile tilapia.

## Data Availability

The authors state the availability of the data.
